# Socio-demographic disparities in knowledge, practices, and ability to comply with COVID-19 public health measures in Canada

**DOI:** 10.17269/s41997-021-00501-y

**Published:** 2021-03-24

**Authors:** Gabrielle Brankston, Eric Merkley, David N. Fisman, Ashleigh R. Tuite, Zvonimir Poljak, Peter J. Loewen, Amy L. Greer

**Affiliations:** 1 Department of Population Medicine, University of Guelph, Guelph, Canada; 2 Munk School of Global Affairs & Public Policy, University of Toronto, Toronto, Canada; 3 Dalla Lana School of Public Health, University of Toronto, Toronto, Canada

**Keywords:** COVID-19, Attitude, Behaviour, Risk perception, Survey, COVID-19, attitude, comportement, perception du risque, enquête

## Abstract

**Objectives:**

The effectiveness of public health interventions for mitigation of the COVID-19 pandemic depends on individual attitudes, compliance, and the level of support available to allow for compliance with these measures. The aim of this study was to describe attitudes and behaviours towards the Canadian COVID-19 public health response, and identify risk-modifying behaviours based on socio-demographic characteristics.

**Methods:**

A cross-sectional online survey was administered in May 2020 to members of a paid panel representative of the Canadian population by age, gender, official language, and region of residence. A total of 4981 respondents provided responses for indicators of self-reported risk perceptions, attitudes, and behaviours towards COVID-19 public health measures.

**Results:**

More than 90% of respondents reported confidence in the ability to comply with a variety of public health measures. However, only 51% reported preparedness for illness in terms of expectation to work if sick or access to paid sick days. Risk perceptions, attitudes, and behaviours varied by demographic variables. Men, younger age groups, and those in the paid workforce were less likely to consider public health measures to be effective, and had less confidence in their ability to comply. Approximately 80% of respondents reported that parents provided childcare and 52% reported that parents in the workforce provided childcare while schools were closed.

**Conclusion:**

Policies to help address issues of public adherence include targeted messaging for men and younger age groups, social supports for those who need to self-isolate, changes in workplace policies to discourage presenteeism, and provincially co-ordinated masking and safe school policies.

**Supplementary Information:**

The online version contains supplementary material available at 10.17269/s41997-021-00501-y.

## Résumé

**Objectifs:**

L’efficacité des mesures d’intervention en santé publique pour atténuer la pandémie de COVID-19 dépend des attitudes individuelles, de la conformité, ainsi que du niveau d’aide disponible pour que les mesures soient respectées. Notre étude visait à décrire les attitudes et les comportements à l’égard de la riposte de la santé publique canadienne à la COVID-19 et à cerner les comportements modificateurs du risque d’après les caractéristiques sociodémographiques.

**Méthode:**

Un sondage en ligne transversal a été administré en mai 2020 aux membres d’un comité rémunéré représentatif de l’âge, du sexe, des langues officielles et des régions de résidence de la population canadienne. En tout, 4 981 personnes ont fourni des réponses à des questions indicatrices de leurs perceptions du risque, de leurs attitudes et de leurs comportements autodéclarés à l’égard des mesures de santé publique liées à la COVID-19.

**Résultats:**

Plus de 90 % des répondants se sont dits certains de leur capacité de respecter de nombreuses mesures de santé publique. Par contre, 51 % seulement ont dit être préparés à respecter ces mesures s’ils attrapaient la maladie, c’est-à-dire pouvoir s’absenter du travail ou avoir droit à des congés de maladie payés. Les perceptions du risque, les attitudes et les comportements variaient selon les caractéristiques démographiques. Les hommes, les jeunes et les personnes ayant un emploi rémunéré étaient moins susceptibles de trouver les mesures de santé publique efficaces et moins sûrs de leur capacité de les respecter. Environ 80 % des répondants ont indiqué que la garde des enfants était assurée par les parents, et 52 % ont indiqué que la garde des enfants quand les écoles étaient fermées était assurée par des parents ayant un emploi.

**Conclusion:**

Des messages ciblant les hommes et les jeunes, des soutiens sociaux aux personnes ayant besoin de s’isoler, des changements dans les politiques en milieu de travail pour dissuader le présentéisme, ainsi que des politiques de port du masque et de sécurité à l’école coordonnées à l’échelle provinciale sont des mesures susceptibles d’atténuer les problèmes d’adhésion du public.

## Introduction

The current coronavirus (COVID-19) pandemic represents a unique challenge for public health and health care systems. In the absence of effective medical treatment options or a safe and effective vaccine, public health agencies have relied on non-pharmaceutical interventions (NPIs) to mitigate transmission of the virus. Physical distancing interventions act to reduce the person-to-person contact rate in a population thereby reducing the likelihood of disease transmission. All Canadian provinces and territories have instituted aggressive physical distancing measures in response to the COVID-19 pandemic, including school closures, remote work, cancellation of mass gatherings, and the closure of all non-essential businesses.

Human behaviour is the main driver of respiratory disease transmission and in the absence of a vaccine or other pharmaceutical interventions, mitigation requires large-scale behaviour change. As such, the effectiveness of public health interventions depends on the level of individual compliance and ability to comply. Perceived risk due to COVID-19 and attitudes toward these measures have a large impact on the willingness of people to make the behaviour changes necessary for public health measures to be effective (Qazi et al. 2020). Perhaps more importantly, the ability to comply with public health measures such as self-isolation is dependent on an individual’s social and financial resources (Atchison et al. 2021).

It is imperative that evidence be used to drive decision-making in order to provide the messaging and supports necessary to minimize transmission. It is important to identify groups who are less likely to perceive COVID-19 as a risk, to perceive that public health measures are effective, and to have the resources to comply, and more likely to engage in behaviours associated with transmission of COVID-19. This information can be used to target messaging, develop policies, and provide supports to encourage uptake of the necessary public health measures. Thus, the objectives of this study are to (1) describe attitudes and behaviours towards the Canadian COVID-19 public health response in May 2020, and (2) identify risk-modifying behaviours and resources to comply with public health measures based on socio-demographic and household characteristics. This paper presents an initial descriptive portrait of attitudes and perceptions among Canadians in the early stage of the pandemic to provide insights into improvements of programs and policies.

## Methods

### Data collection

The study protocol was approved by the University of Guelph Research Ethics Board (protocol #20-04-011) and the University of Toronto Research Ethics Board (protocol #38251). The research company, Dynata, was contracted to conduct an electronic survey of Canadian adults between May 7 and May 19, 2020. Participants were recruited from a panel of survey respondents and paid a nominal amount for completing the survey. Panelists who logged into their Dynata account during the study period were directed to the survey if they fit the quotas being targeted. Respondents provided informed consent after reading the study information by choosing to continue to the survey questions. Representativeness of the survey sample population was ensured by setting quotas on age, gender, official language (English and French), and region of residence (i.e., Atlantic, Quebec, Ontario, and West) based on 2016 Canadian Census data (Statistics Canada 2016a). Respondents could complete the survey in English or French. Enrollment into the survey within each stratum was on a first-come, first-served basis.

The survey instrument was adapted from previous work (Jarvis et al. 2020) and posed questions about perceived risk of COVID-19 infection, as well as attitudes and behaviours regarding COVID-19 public health measures. Participants provided information about their age, gender, province of residence, education level, employment status, household composition, household income, and the general size of their location of residence with options ranging from large city to rural. Participants were also asked whether they would be considered a priority risk group to receive the seasonal influenza vaccine as outlined by the Public Health Agency of Canada. The conditions meeting this criterion included chronic respiratory disease, chronic heart disease, chronic kidney disease, chronic liver disease, chronic neurological disease, diabetes (all types), cancer, immunosuppression, dysfunction of the spleen, and/or BMI > 40 (National Advisory Committee on Immunization (NACI) 2019).

COVID-19 risk perceptions were assessed by 3 statements and each response was recorded using a 6-level Likert scale ranging from strongly agree to strongly disagree, and unsure. Perceived effectiveness of public health interventions to control COVID-19 was assessed by 8 questions, the responses to which were recorded using a 5-level Likert scale ranging from very effective to not at all effective, and unsure. Respondents’ confidence that they could comply with various public health measures related to COVID-19 was assessed by 7 items and responses were recorded using a 5-level Likert scale ranging from very confident to not at all confident, and unsure. Ability to comply with public health measures due to external influences was assessed using a 5-item Likert scale, ranging from strongly agree to strongly disagree.

Participants were also asked about their use of face masks. Adults living with children under the age of 15 years were asked to provide information about childcare provision during school and daycare closures. The complete survey is provided in the Supplementary Materials.

### Data analysis

Demographic characteristics of survey respondents were compared with those from the 2016 Canadian Census in order to ensure that the sample population was generally representative of the Canadian population.

Attitudes towards the effectiveness of COVID-19 measures and confidence in individuals’ ability to comply with such measures were aggregated to provide binary measures of agreement (strongly or somewhat); confidence (very or fairly); and perceived effectiveness of measures (very or fairly); with the other category comprised of neutral responses, non-agreement, or uncertainty. For a question regarding expectations of coworkers with respect to working while ill, the responses “somewhat disagree” and “strongly disagree” were combined to form “disagree”, while all other responses were combined. The proportion of respondents who agreed, were confident, or thought each measure was effective was calculated for each of the questions about attitudes toward COVID-19 public health measures. Chi-square analyses of individual contingency tables were conducted to further explore these data by respondent demographics and household characteristics. The Bonferroni correction was applied for each of the indicators of attitudes toward and ability to comply with public health measures to account for multiple comparisons within each measure. Therefore, a relationship was considered significant if the *p*-value was less than the corrected value (0.05/19 = 0.0026). Pairwise post hoc analyses with a Bonferroni correction were conducted for multi-level demographic and household variables which showed significance in the chi-square analyses.

Logistic regression models were developed to identify factors associated with (1) mask use in the 24 hours prior to survey completion, (2) reporting direct contact with individuals outside of the respondent’s immediate household in the 7 days prior to survey completion, and (3) reporting confidence in the ability to self-isolate with mild symptoms of COVID-19. Univariable models were first assessed using a liberal *p*-value of less than 0.3 to determine eligibility for inclusion in the multivariable models. Variables included in the initial full model for mask use included age, gender, risk group status, size of geographic region of residence, household income, education level, employment status, household composition, and household size, as well as two indicators of perceived risk of COVID-19 to self and one indicator of perceived risk of transmission to others. The initial full model assessing factors associated with engaging in an activity with non-household contacts included respondents’ perceived effectiveness of reducing contacts to mitigate transmission in addition to all variables included in the model described above for mask use. The initial full model for confidence in the ability to self-isolate included demographic characteristics and indicators of perceived risk as above in addition to workplace indicators such as access to paid sick leave, expectation to work while sick, and the ability to work remotely. Household characteristics such as household size, whether the household included children or a single parent, and whether the household had access to a 14-day stockpile of supplies were also included in the initial model. Further details of this regression model are located in the Supplementary Materials.

A backward elimination procedure was used to evaluate variables for inclusion in the final multivariable regression models. Confounding was assessed by examining the variables in the model for changes once the potentially confounding variable was excluded from the model. Once the final model was identified, all two-way multiplicative interaction terms involving age group with the other predictor variables were assessed. Age group was of interest because it was significantly associated with most measures of perceived effectiveness and ability to comply with public health measures. Multicollinearity was assessed for each model using the variance inflation factor. All data were analyzed using RStudio Version 1.2.5033 (RStudio Team 2019).

## Results

A total of 9120 survey responses were received between May 7 and May 19, 2020. Survey responses were excluded from analysis if the survey was completed in less than one third of the estimated completion time (*n* = 137), if the respondent reported their age as less than 18 years (*n* = 23), or if the survey was discontinued prior to completion for any reason, including exceeding the age, gender, or province quotas (*n* = 3960). Responses with duplicated entries for gender, age, postal code, date, and contact names were considered duplicate responses and removed from the dataset (*n* = 19). Respondents who completed the entire survey and were not screened out for any reason were included in the final sample, resulting in 4981 high-quality survey responses.

A detailed description of the respondent population is included in the Supplementary Materials (Table S1). For the 4981 surveys, the proportion of respondents living in each province, the male to female ratio, and the proportion of respondents in each age category were comparable to the 2016 Canadian Census of the population (data shown in Table S1, Supplementary Materials).

### Perceived risk

Table 1 describes respondents’ level of perceived risk in May 2020 as well as indicators of preparedness in the event of illness. Overall, 61.5% of respondents agreed that COVID-19 would be a serious illness for them, 21.5% agreed that they are likely to catch the virus, and 71.5% agreed that they are likely to transmit the virus if they do not follow public health advice. Perceived risk of serious illness due to COVID-19 increased with increasing age beyond 50 years; however, perceived risk of contracting the virus was highest in the 30–39 year age group and decreased with increasing age thereafter. Individuals who self-identified as being in a risk group were more likely to agree that they are likely to catch the virus and experience serious illness compared with other individuals, while those living with children under the age of 18 years or those in the paid workforce were less likely to agree that COVID-19 would be a serious illness for them compared with households containing no children or those not in the paid workforce, respectively. Risk perception was also associated with gender (Table 1).

**Table 1:** Indicators of perceived risk and preparedness in the event of illness stratified by socio-demographic characteristics. Values are reported as % (95% confidence interval), and those in bold font were statistically significant between subgroups (*p* < 0.0026). Cells denoted by “-” signify that statistics were not run because the survey question was not relevant for one of the groups. Letters in superscript indicate levels at which pairwise comparisons were statistically significant with Bonferroni correction (*p* < 0.05)

	Perceived risk			Preparedness in the event of illness			
	Likely to contract COVID-19 (*n* = 4981)	Likely to be a serious illness for self (*n* = 4981)	Likely to transmit to others (*n* = 4981)	My co-workers would not expect me to work if ill (*n* = 2698)	I would still get paid (*n* = 2698)	I have enough food/supplies to last for 14 days (*n* = 4981)	Someone else would be able to look after my children (*n* = 930)
Overall	21.5% (20.4–22.7)	61.5% (60.1–62.8)	71.5% (70.3–72.8)	50.9% (49.0–52.7)	51.1% (49.2–53.0)	70.8% (69.6–72.1)	59.6% (56.4–62.7)
Gender							
Women	20.8% (19.3–22.4)	61.0% (59.1–62.9)	**74.5% (72.8–76.2)**	**55.7% (52.9–58.4)**	48.7% (45.9–51.5)	**72.9% (71.2–74.7)**	**53.2% (48.7–57.6)**
Men	22.1% (20.5–23.8)	61.9% (60.0–63.8)	**68.4% (66.5–70.2)**	**46.8% (44.2–49.3)**	53.3% (50.7–55.9)	**68.8% (66.9–70.6)**	**66.2% (61.9–70.6)**
Age group							
18–29 years^a^	**25.7%** ^**d–f**^ **(22.7–28.8)**	**45.1%** ^**c–f**^ **(41.6–48.6)**	70.9% (67.8–74.1)	**40.0%** ^**c, d**^ **(35.6–44.3)**	**48.2%** ^**b**^ **(43.8–52.7)**	**62.5%** ^**c–f**^ **(59.1–65.9)**	**54.4%** ^**b, c, e**^ **(46.0–62.7)**
30–39 years^b^	**32.2%** ^**c–f**^ **(29.2–35.2)**	**51.6%** ^**d–f**^ **(48.4–54.8)**	71.4% (68.6–74.3)	**43.3%** ^**c, d**^ **(39.8–46.8)**	**55.3%** ^**a, e**^ **(51.7–58.8)**	**65.6%** ^**e, f**^ **(62.5–68.6)**	**56.7%** ^**a, e**^ **(51.6–61.9)**
40*–*49 years^c^	**24.3%** ^**b, e, f**^ **(21.4–27.3)**	**54.9%** ^**a, d–**^^**f**^ **(51.4–58.3)**	70.3% (67.2–73.5)	**52.4%** ^**a, b**^ **(48.5–56.2)**	**54.1%** ^**e**^ **(50.2–58.0)**	**66.5%** ^**a, e, f**^ **(63.2–69.7)**	**60.7%** ^**a, d, e**^ **(55.3–66.1)**
50*–*59 years^d^	**18.4%** ^**a, b**^ **(15.8–21.0)**	**62.6%** ^**a-c, e, f**^ **(59.4–65.9)**	71.3% (68.3–74.4)	**63.4%** ^**a,b, e**^ **(59.4–67.4)**	**50.9%** ^**e**^ **(46.7–55.1)**	**71.6%** ^**a, e, f**^ **(68.5–74.6)**	**77.8%** ^**c, e**^ **(69.2–86.4)**
60*–*69 years^e^	**13.4%** ^**a–c**^ **(11.3–15.6)**	**72.9%** ^**a–c, d, f**^ **(70.1–75.7)**	70.6% (67.8–73.5)	**62.0% (55.9–68.2)**	**41.4%** ^**b–d**^ **(35.1–47.6)**	**77.8%** ^**a–d**^ **(75.2–80.4)**	**35.0%** ^**a, b, d**^ **(14.1–55.9)**
70+ years^f^	**13.8%** ^**a–c**^ **(11.1–16.5)**	**85.3%** ^**a–d**^ **(82.5–88.0)**	75.6% (72.2–78.9)	**-**	**-**	**82.5%** ^**a–d**^ **(79.5–85.4)**	-
Risk group							
Yes	**29.8% (27.6–32.0)**	**86.1% (84.4–87.7)**	74.0% (71.9–76.2)	**44.0% (40.3–47.6)**	52.7% (49.1–56.4)	**73.6% (71.5–75.8)**	63.4% (57.5–69.3)
No	**17.5% (16.2–18.8)**	**49.6% (47.9–51.3)**	70.3% (68.8–71.9)	**53.4% (51.2–55.6)**	50.5% (48.3–52.7)	**69.4% (67.9–71.0)**	58.1% (54.4–61.9)
Household children							
Yes	**29.0% (26.4–31.7)**	**55.8% (52.8–58.7)**	71.0% (68.3–73.6)	48.3% (45.0–51.7)	53.4% (50.1–56.8)	**66.1% (63.4–68.9)**	-
No	**19.3% (18.1–20.6)**	**63.1% (61.6–64.7)**	71.7% (70.3–73.1)	52.0% (50.0–54.3)	50.0% (47.7–52.3)	**72.2% (70.8–73.6)**	-
Household income							
$0–$60,000^g^	21.8% (20.0–23.7)	**64.8%** ^**h**^ **(62.6–66.9)**	70.3% (68.3–72.4)	51.0% (47.4–54.6)	**40.0%** ^**h, i**^ **(36.4–43.5)**	68.0% (65.9–70.1)	**49.3%** ^**h, i**^ **(42.8–55.8)**
$60,001–$110,000^h^	22.9% (20.8–25.0)	**58.5%** ^**g**^ **(56.0–60.9)**	73.0% (70.8–75.2)	51.6% (48.5–54.7)	**55.0%** ^**g, i**^ **(51.9–58.1)**	73.5% (71.3–75.6)	**62.2%** ^**g, i**^ **(57.2–67.2)**
> $110,000^i^	22.4% (19.8–24.9)	**60.5% (57.5–63.4)**	73.1% (70.4–75.9)	48.2% (44.7–51.7)	**57.8%** ^**g, h**^ **(54.3–61.3)**	71.6% (68.8–74.3)	**65.1%** ^**g, h**^ **(59.6–70.7)**
Don’t know/prefer not to answer	14.5% (11.4–17.5)	**60.6% (56.3–64.8)**	68.4% (64.3–72.4)	57.0% (50.1–63.9)	**47.0% (40.1–53.9)**	71.3% (67.4–75.2)	**56.5% (44.1–68.8)**
Employment status							
Paid workforce	**26.2% (24.6–27.9)**	**56.3% (54.4–58.1)**	70.6% (68.9–72.3)	-	-	**68.7% (66.9–70.4)**	61.5% (57.9–65.1)
Not in paidworkforce	**16.0% (14.5–17.5)**	**67.6% (65.7–69.5)**	72.6% (70.8–74.5)	-	-	**73.3% (71.5–75.1)**	53.1% (46.4–59.8)
Level of education							
Secondary or less^j^	18.7% (16.5–20.9)	61.7% (59.0–64.5)	71.1% (68.5–73.6)	53.6% (48.9–58.2)	**40.3%** ^**k, l**^ **(35.7–44.9)**	71.4% (68.8–74.0)	50.3% (42.3–58.4)
College/trades or other qualification^k^	21.7% (19.9–23.6)	62.1% (59.9–64.3)	70.0% (67.9–72.1)	51.5% (48.4–54.6)	**50.7%** ^**j, l**^ **(47.6–53.8)**	71.4% (69.3–73.4)	58.2% (53.0–63.4)
University degree^l^	23.1% (21.2–25.0)	60.6% (58.4–62.9)	73.4% (71.4–75.4)	49.5% (46.7–52.2)	**55.1%** ^**j**^ **(52.4–57.9)**	69.9% (67.8–72.0)	63.8% (59.3–68.4)

### Perceived preparedness

A higher proportion of older individuals, those not in a risk group, and women reported that co-workers would not expect them to work if sick (Table 1). Fewer respondents in the youngest age group (18–29 years) reported having access to paid sick leave compared with those in the 30–39 years age group, and those aged 30–59 years were more likely to have access to paid sick leave compared with respondents aged 60–69 years (Table 1). Demographics also predicted confidence in access to a 14-day supply of food, and ability to find childcare (Table 1).

### Perceived effectiveness and confidence in the ability to comply with public health measures

At least 87% of respondents considered each of the public health measures described to be effective in reducing the transmission of COVID-19, with women and older individuals expressing greater faith in public health measures (Table 2). Those in the paid workforce were less likely to agree that each of the public health measures is effective except school closures, where there was no difference between groups.

**Table 2:** Perceived effectiveness of six different public health measures stratified by socio-demographic characteristics. Values are reported as % (95% confidence interval), and those in bold font were statistically significant between subgroups (*p* < 0.0026) (*n* = 4981). Letters in superscript indicate levels at which pairwise comparisons were statistically significant with Bonferroni correction (*p* < 0.05)

	Public health measures					
	Reduce contacts	Self-isolate for 14 days with severe respiratory symptoms	Avoid crowds	Stay home for 14 days when household member has severe respiratory symptoms	School closures	Business closures
Overall	93.7% (93.0–94.3)	93.1% (92.4–93.8)	94.0% (93.3–94.6)	90.6% (89.8–91.4)	87.2% (86.2–88.1)	91.4% (90.6–92.2)
Gender						
Women	**95.3% (94.4–96.1)**	**94.9% (94.0–95.7)**	**95.8% (95.0–96.6)**	**92.5% (91.5–93.5)**	**89.6% (88.5–90.8)**	**93.4% (92.4–94.4)**
Men	**92.0% (91.0–93.1)**	**91.3% (90.2–92.4)**	**92.1% (91.1–93.2)**	**88.8% (87.5–90.0)**	**84.7% (83.2–86.1)**	**89.4% (88.1–90.6)**
Age group						
18–29 years^a^	**90.4%** ^**c–f**^ **(88.3–92.5)**	**89.9%** ^**e, f**^ **(87.8–92.0)**	**89.8%** ^**d–f**^ **(87.7–91.9)**	**86.2%** ^**d–f**^ **(83.8–88.6)**	85.8% (83.3–88.2)	**87.1%** ^**e, f**^ **(84.7–89.4)**
30–39 years^b^	**92.0%** ^**e, f**^ **(90.2–93.7)**	**91.2%** ^**e, f**^ **(89.4–93.0)**	**91.4%** ^**d–f**^ **(89.7–93.2)**	**88.2%** ^**e, f**^ **(86.2–90.3)**	86.7% (84.6–88.9)	**88.9%** ^**e, f**^ **(86.9–90.9)**
40–49 years^c^	**91.3%** ^**a, e, f**^ **(89.3–93.2)**	**92.5% (90.7–94.3)**	**92.3%** ^**e, f**^ **(90.4–94.1)**	**90.9% (88.9–92.9)**	85.8% (83.4–88.2)	**90.8%** ^**e, f**^ **(88.8–92.8)**
50–59 years^d^	**94.8%** ^**a, f**^ **(93.3–96.3)**	**93.9%** **(92.3–95.5)**	**95.4%** ^**a, b**^ **(94.0–96.8)**	**92.2%** ^**a**^ **(90.4–94.0)**	88.1% (85.9–90.3)	**91.4%** ^**e, f**^ **(89.5–93.3)**
60–69 years^e^	**95.9%** ^**a–c**^ **(94.7–97.2)**	**95.4%** ^**a, b**^ **(94.1–96.7)**	**97.1%** ^**a–c**^ **(96.1–98.2)**	**92.7%** ^**a, b**^ **(91.1–94.4)**	88.2% (86.2–90.3)	**95.0%** ^**a–d**^ **(93.6–96.4)**
70+ years^f^	**98.1%** ^**a –d**^ **(97.1–99.2)**	**95.8%** ^**a, b**^ **(94.2–97.3)**	**98.1%** ^**a–c**^ **(97.1–99.2)**	**93.9%** ^**a, b**^ **(92.0–95.8)**	88.4% (85.9–90.9)	**95.8%** ^**a–d**^ **(94.2–97.3)**
Risk group						
Yes	**95.3% (94.2–96.3)**	93.4% (92.2–94.6)	94.1% (92.9–95.2)	90.3% (88.8–91.7)	88.8% (87.2–90.3)	92.8% (91.6–94.1)
No	**92.9% (92.0–93.8)**	92.9% (92.1–93.8)	93.9% (93.1–94.7)	90.8% (89.8–91.8)	86.4% (85.2–87.6)	90.7% (89.7–91.7)
Household children						
Yes	92.6% (91.1–94.1)	93.1% (91.6–94.5)	93.1% (91.6–94.5)	90.4% (88.7–92.1)	87.6% (85.7–89.5)	90.2% (88.4–91.9)
No	94.0% (93.2–94.7)	93.1% (92.3–93.9)	94.2% (93.5–95.0)	90.7% (89.8–91.6)	87.0% (86.0–88.1)	91.8% (90.9–92.7)
Household income						
$0–$60,000	93.2% (92.0–94.3)	92.9% (91.7–94.1)	94.3% (93.3–95.4)	90.2% (88.9–91.6)	87.8% (86.3–89.2)	91.6% (90.3–92.8)
$60,001–$110,000	94.9% (93.8–96.0)	94.8% (93.7–95.9)	94.9% (93.8–96.0)	92.4% (91.1–93.8)	88.2% (86.6–89.8)	93.1% (91.9–94.4)
> $110,000	92.5% (90.9–94.1)	91.5% (89.8–93.2)	92.0% (90.3–93.7)	89.4% (87.5–91.2)	85.1% (82.9–87.2)	88.7% (86.7–90.6)
Don’t know/prefer not to answer	94.0% (91.9–96.0)	91.6% (89.2–94.0)	93.8% (91.7–95.9)	89.1% (86.4–91.8)	86.1% (83.1–89.1)	91.0% (88.5–93.5)
Employment status						
Paid workforce	**92.6% (91.6–93.5)**	92.2% (91.2–93.2)	**92.6% (91.6–93.6)**	**89.3% (88.2–90.5)**	86.6% (85.3–87.9)	**89.4% (88.2–90.6)**
Not in paid workforce	**95.0% (94.1–95.9)**	94.1% (93.2–95.1)	**95.6% (94.7–96.4)**	**92.2% (91.1–93.3)**	87.9% (86.5–89.2)	**93.8% (92.8–94.8)**
Level of education						
Secondary or less	93.0% (91.5–94.4)	92.7% (91.2–94.2)	93.8% (92.4–95.2)	90.9% (89.2–92.5)	88.1% (86.3–89.9)	91.4% (89.8–93.0)
College/trades or other qualification	93.7% (92.6–94.8)	93.5% (92.4–94.6)	93.8% (92.8–94.9)	90.2% (88.9–91.5)	86.3% (84.7–87.8)	90.5% (89.2–91.8)
University degree	94.0% (93.0–95.1)	92.9% (91.8–94.1)	94.2% (93.1–95.2)	90.9% (89.6–92.2)	87.5% (86.0–89.0)	92.3% (91.1–93.5)

More than 90% of respondents reported that they were confident in their ability to comply with each of the five public health measures (Table 3), with greater confidence on most measures in women and older individuals. Lower-income individuals were less confident in their ability to avoid public transportation. Less confidence was seen in the paid workforce, compared with those who were unemployed, retired, or working within the home.

**Table 3:** Confidence in the ability to comply with five different public health measures stratified by socio-demographic characteristics. Values are reported as % (95% confidence interval), and those in bold font were statistically significant between subgroups (*p* < 0.0026) (*n* = 4981). Letters in superscript indicate levels at which pairwise comparisons were statistically significant with Bonferroni correction (*p* < 0.05)

Public health measures					
	Reduce contacts	Self-isolate for 14 days with severe respiratory symptoms	Avoid crowds	Stay home for 14 days when household member has severe respiratory symptoms	Avoid public transportation
Overall	93.7% (93.0–94.4)	93.0% (92.3–93.7)	93.3% (92.6–94.0)	91.0% (90.2–91.7)	90.9% (90.0–91.7)
Gender					
Women	94.6% (93.7–95.5)	**94.9% (94.1–95.8)**	**94.7% (93.8–95.6)**	**92.7% (91.6–93.7)**	**92.9% (91.9–93.9)**
Men	92.8% (91.8–93.9)	**91.1% (90.0–92.2)**	**91.9% (90.8–92.9)**	**89.3% (88.1–90.5)**	**88.8% (87.6–90.1)**
Age group					
18–29 years ^a^	**90.1%** ^**c–f**^ **(88.1–92.2)**	**87.7%** ^**c–f**^ **(85.4–90.0)**	**88.1.%** ^**c–f**^ **(85.8–90.4)**	**85.6%** ^**c–f**^ **(83.2–88.1)**	**85.3%** ^**c–f**^ **(82.8–87.8)**
30–39 years ^b^	**92.6%** ^**e**^ **(90.9–94.3)**	**90.6%** ^**e, f**^ **(88.7–92.5)**	**89.2%** ^**c–f**^ **(87.2–91.2)**	**87.5%** ^**c–f**^ **(85.4–89.6)**	**86.8%** ^**c–f**^ **(84.7–89.0)**
40–49 years ^c^	**92.0%** ^**a, e, f**^ **(90.1–93.9)**	**92.1%** ^**a, e, f**^ **(90.3–94.0)**	**91.8%** ^**a, c–f**^ **(89.9–93.7)**	**89.7%** ^**a, c–f**^ **(87.5–91.8)**	**89.4%** ^**a, b, e, f**^ **(87.3–91.5)**
50–59 years ^d^	**94.3%** ^**a**^ **(92.8–95.9)**	**94.2%** ^**a**^ **(92.7–95.8)**	**95.6%** ^**a**^ **(94.3–97.0)**	**93.9%** ^**a**^ **(92.3–95.5)**	**92.1%** ^**a, b, f**^ **(90.3–93.9)**
60–69 years ^e^	**96.7%** ^**a–c**^ **(95.6–97.8)**	**96.4%** ^**a–c**^ **(95.3–97.6)**	**97.2%** ^**a**^ **(96.2–98.3)**	**94.2%** ^**a**^ **(92.7–95.6)**	**95.0%** ^**a–c**^ **(93.6–96.4)**
70+ years ^f^	**96.1%** ^**a, c**^ **(94.6–97.6)**	**97.2%** ^**a–c**^ **(95.9–98.5)**	**98.4%** ^**a**^ **(97.5–99.4)**	**95.3%** ^**a**^ **(93.7–97.0)**	**97.3%** ^**a–c**^ **(96.1–98.6)**
Risk group					
Yes	95.1% (94.0–96.1)	92.8% (91.5–94.0)	94.3% (93.1–95.4)	91.0% (89.6–92.4)	91.4% (90.1–92.8)
No	93.0% (92.2–93.9)	93.1% (92.2–94.0)	92.8% (92.0–93.7)	90.9% (90.0–91.9)	90.6% (89.6–91.6)
Household children					
Yes	92.6% (91.1–94.1)	91.6% (90.0–93.3)	91.7% (90.1–93.3)	90.0% (88.2–91.7)	89.2% (87.4–91.0)
No	94.0% (93.2–94.7)	93.4% (92.6–94.2)	93.8% (93.0–94.5)	91.2% (90.3–92.1)	91.3% (90.5–92.2)
Household income					
$0–$60,000 ^g^	93.2% (92.0–94.3)	92.6% (91.4–93.8)	93.3% (92.1–94.4)	90.2% (88.8–91.5)	**89.3%** ^**h**^ **(87.9–90.7)**
$60,001–$110,000 ^h^	94.7% (93.6–95.8)	94.5% (93.4–95.7)	94.0% (92.8–95.1)	92.0% (90.7–93.3)	**93.0%** ^**g**^ **(91.7–94.2)**
> $110,000	93.8% (92.3–95.2)	91.7% (90.0–93.4)	92.7% (91.1–94.3)	90.4% (88.6–92.2)	**90.8% (89.1–92.6)**
Don’t know/prefer not to answer	92.2% (89.9–94.5)	92.4% (90.1–94.7)	92.6% (90.3–94.9)	91.6% (89.2–94.0)	**90.2% (87.7–92.8)**
Employment status					
Paid workforce	**92.4% (91.4–93.4)**	**91.4% (90.3–92.4)**	**91.1% (90.1–92.2)**	**88.9% (87.7–90.1)**	**88.9% (87.7–90.1)**
Not in paid workforce	**95.2% (94.3–96.1)**	**94.9% (94.0–95.8)**	**95.8% (95.0–96.7)**	**93.4% (92.4–94.4)**	**93.2% (92.1–94.2)**
Level of education					
Secondary or less	92.7% (91.2–94.2)	92.5% (91.0–94.0)	93.1% (91.7–94.6)	90.1% (88.4–91.8)	90.4% (88.7–92.0)
College/trades or other qualification	93.6% (92.5–94.7)	93.6% (92.5–94.7)	93.7% (92.6–94.8)	92.2% (91.0–93.4)	91.5% (90.2–92.7)
University degree	94.4% (93.4–95.4)	92.8% (91.6–94.0)	93.0% (91.8–94.1)	90.2% (88.9–91.6)	90.5% (89.2–91.9)

### Childcare

**Fig. 1:**
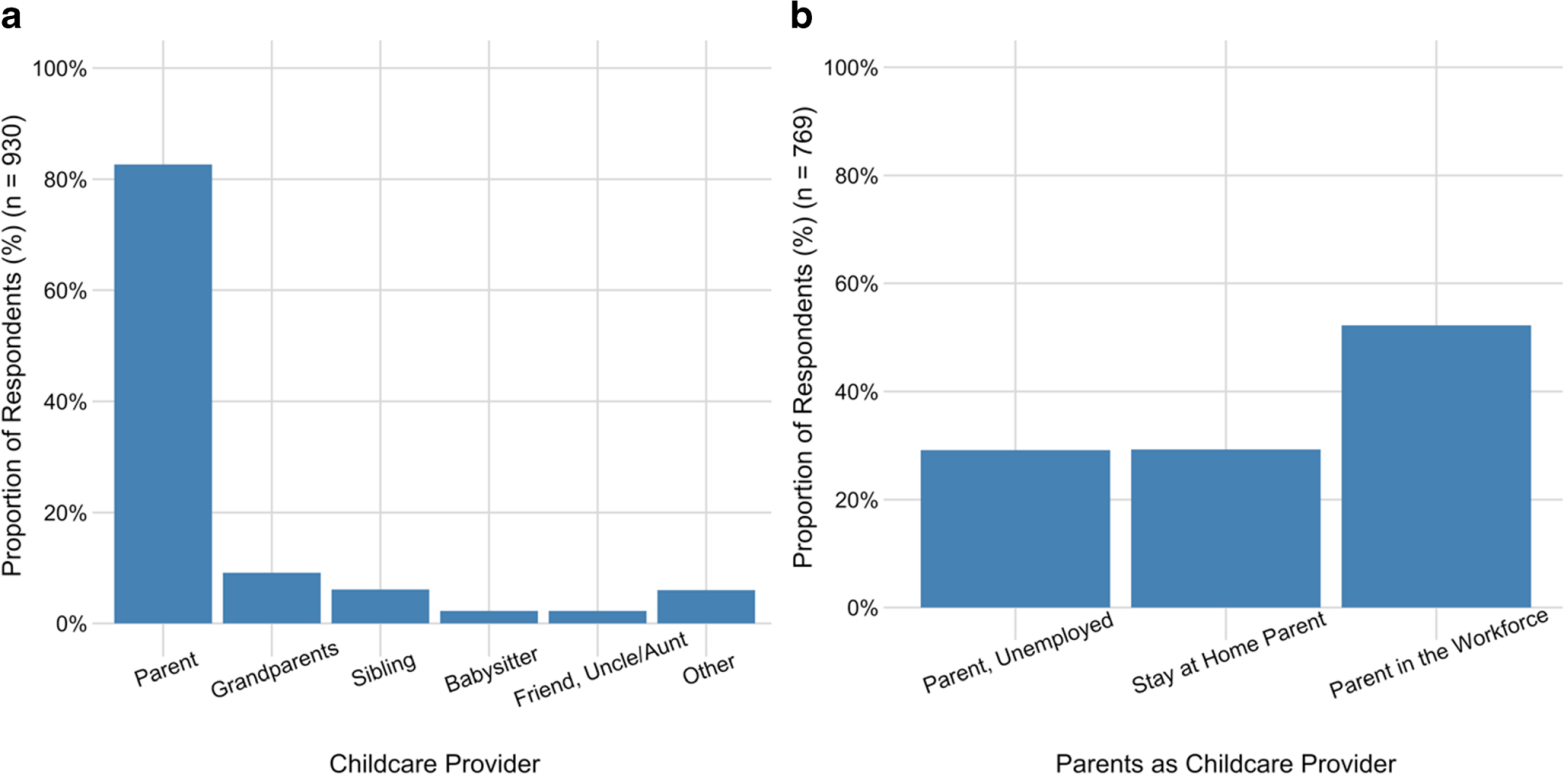
**a** Respondents with children 14 years of age or younger (*n* = 930) reported on which individuals looked after the children in their household during school and daycare closures due to the pandemic. **b** Respondents who reported that parents provided childcare during school and daycare closures (*n* = 769) also identified the employment circumstances of the parent who provided the childcare. The category “Parent in the Workforce” includes those working remotely, those working part-time, those who took leave from their job, and those who were unemployed due to COVID-19 but otherwise have been working

Respondents with household members who were 14 years of age or younger were asked about childcare provision when schools and daycares were closed due to the pandemic (*n* = 930). More than 80% of respondents reported that a parent provided childcare for their children during this time (Fig. 1a). Only 12.2% (95% CI, 10.1–14.3) of those requiring childcare used providers who were not part of their household. Of the parents providing childcare, parents in the workforce provided the greatest proportion of childcare duties (52%) (Fig. 1b). The wording of the questionnaire did not allow for an analysis of childcare by gender.

### Predictors of mask use

**Fig. 2:**
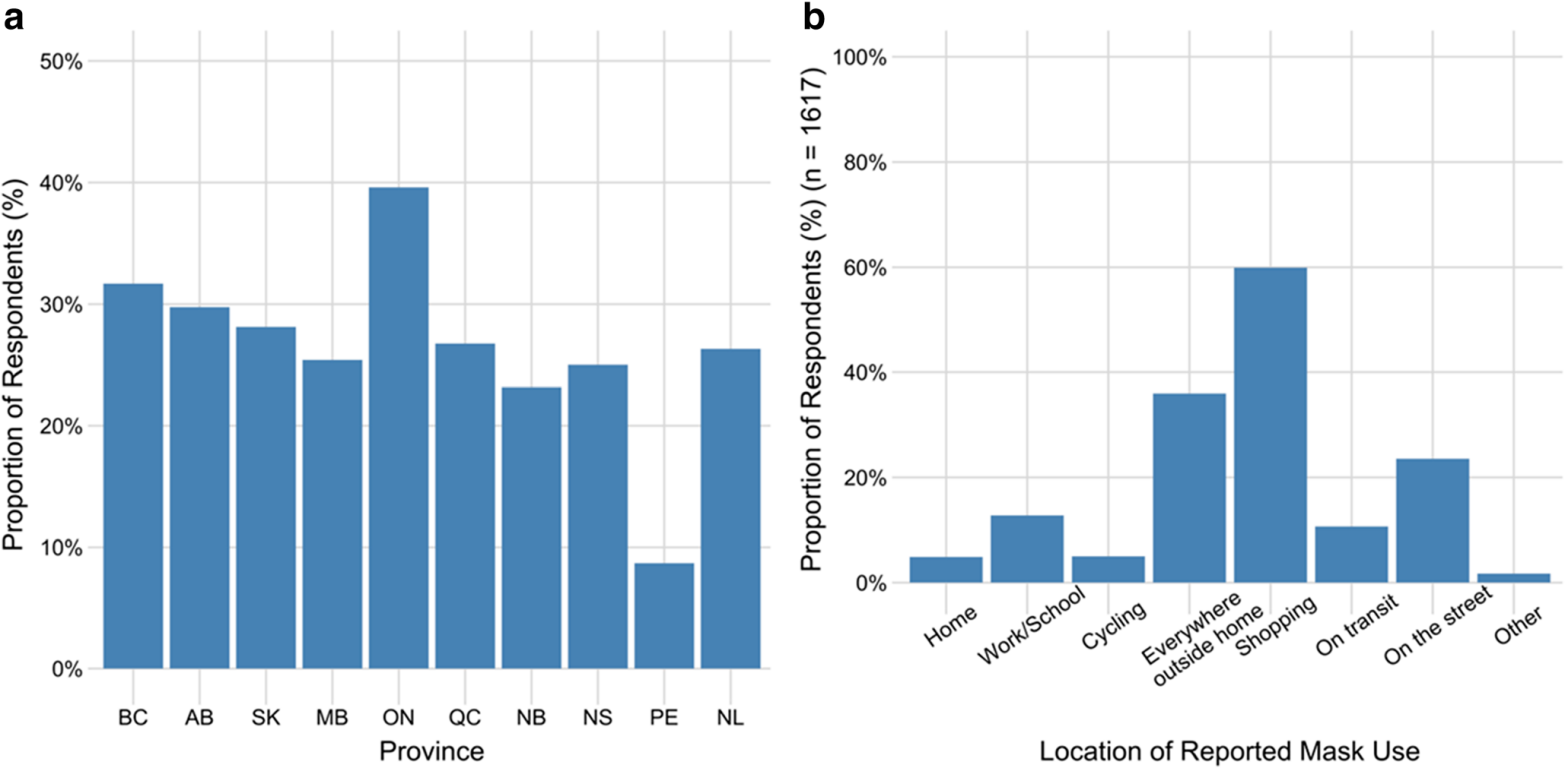
Respondents were asked if they had worn a face mask in the 24 hours prior to survey completion. **a** represents reported mask use by province of residence. **b** identifies the location(s) of mask use for respondents who reported wearing a mask in the previous 24 hours (*n* = 1617)

The proportion of respondents who wore a mask in the 24 hours prior to survey completion was 32.5% (95% CI, 31.2–33.8) for an average duration of 96.6 (SD 412.4) minutes. Respondents from Ontario (where physical distancing measures were still in place at the time of the survey) reported the highest level of mask use while those from Prince Edward Island (where physical distancing recommendations were beginning to relax at the time of the survey) reported the lowest mask use (Fig. 2a). The most common locations to wear a mask were in supermarkets or other stores, anywhere outside the home, and walking on the street (Fig. 2b); 42.7% (95% CI, 35.3–50.1) of mask-wearing transit-users had worn a mask on transit in the past 24 hours.

Factors associated with mask use are shown in Table 4; mask use was increased in households with more than one adult, with children, or with multiple generations; and in individuals with university-level education, or reporting that they would be at risk of serious illness with COVID-19 or at increased risk of developing COVID-19. Of the variables assessed for interaction with age group, the only interaction detected was between age and high-risk conditions with younger (< 30 years), high-risk individuals more likely to have reported mask use compared with 40–49-year-old respondents who were not in a risk group.

**Table 4:** Results of a multivariable logistic regression analysis assessing factors associated with mask use in the 24 hours prior to survey completion. Values are reported as adjusted odds ratios (95% confidence interval) and those in bold font were statistically significant (*p* < 0.05) (*n* = 4981)

Variable	Adjusted OR (95% CI)	*p* Wald’s test	*p* (L-R test)
Household composition			0.005
Single person living alone (referent)			
**Adults only living together**	**1.32 (1.13–1.55)**	**< 0.001**	
**Family with children**	**1.24 (1.01–1.52)**	**0.04**	
**>2 generations living together**	**1.71 (1.12–2.63)**	**0.01**	
Grandparents living with their grandchildren only	1.16 (0.28–4.72)	0.84	
Age category			1
**18–29 years**	**1.61 (1.23–2.10)**	**< 0.001**	
30–39 years	1.21 (0.95–1.56)	0.13	
40–49 years (referent)	-	-	
50–59 years	1.22 (0.93–1.60)	0.15	
**60–69 years**	**1.44 (1.08–1.92)**	**0.01**	
Over 70 years	1.39 (0.98–1.99)	0.07	
Respondent risk group			1
**Respondent is in a high-risk group**	**1.51 (1.06–2.15)**	**0.02**	
Size of the geographic region of residence			< 0.001
Large city (referent)			
**Medium-sized city**	**0.74 (0.64–0.85)**	**< 0.001**	
**Large town**	**0.61 (0.48–0.76)**	**< 0.001**	
**Small town**	**0.53 (0.43–0.66)**	**< 0.001**	
**Rural place**	**0.38 (0.29–0.50)**	**< 0.001**	
Education level of respondent			0.02
Secondary or less (referent)			
College/trade/other qualification	1.00 (0.85–1.18)	1.0	
**University (bachelor degree or higher)**	**1.21 (1.02–1.43)**	**0.03**	
Employment status of respondent			< 0.001
Unemployed, student, retired, work within home (referent)			
**Employed FT, PT, self-employed**	**1.32 (1.13–1.54)**	**< 0.001**	
Perceived risk of contracting the virus			< 0.001
**Likely to contact the virus**	**1.31 (1.12–1.52)**	**< 0.001**	
Perceived risk of serious illness due to COVID-19			< 0.001
**COVID-19 would be a serious illness for respondent**	**1.61 (1.39–1.85)**	**< 0.001**	
Interaction between age category and respondent risk group			< 0.001
**18–29 years in a risk group**	**1.75 (1.06–2.90)**	**0.03**	
30–39 years in a risk group	1.57 (0.98–2.53)	0.06	
40–49 years, not high risk (referent)	-	-	
50–59 years in a risk group	0.87 (0.54–1.39)	0.56	
60–69 years in a risk group	0.72 (0.46–1.13)	0.15	
Over 70 years in a risk group	0.89 (0.54–1.46)	0.64	

### Direct contact with non-household members

**Fig. 3:**
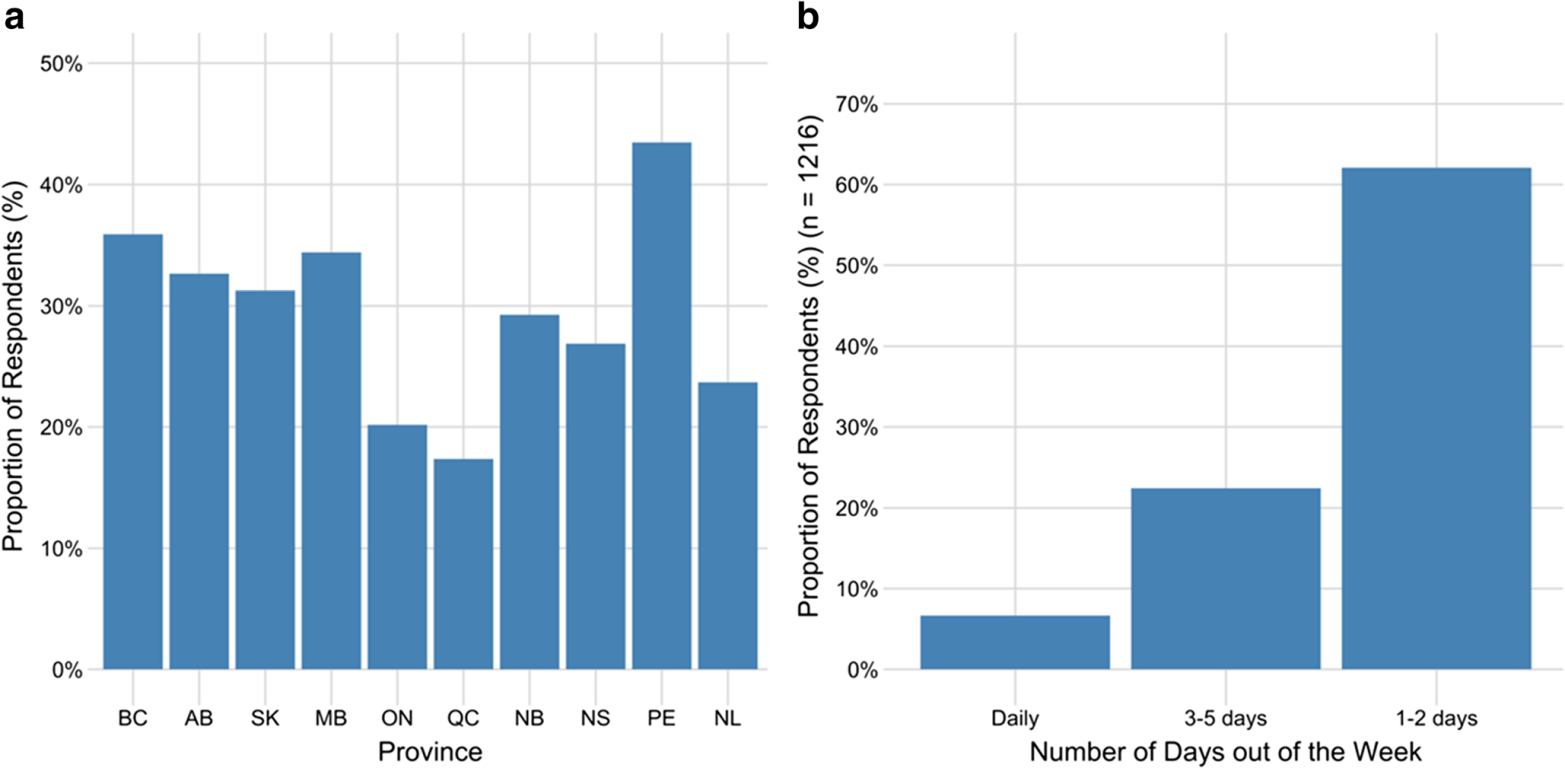
**a** Proportion of respondents reporting contact with non-household members in the 7 days prior to survey completion**.** More non-household contacts were reported for provinces which were more advanced in the de-escalation of physical distancing (e.g., PEI) at the time of survey completion; however, in provinces where physical distancing was still in place during the survey period (e.g., ON), approximately 20% of respondents were reporting non-household contacts in May 2020. **b** The number of days in the past week respondents engaged in an activity with a non-household contact, for those reporting such activity (*n* = 1216)

The proportion of respondents who had engaged in an activity with non-household contacts in the 7 days prior to survey completion was 24.4% (95% CI, 23.2–25.6) (Fig. 3a). More non-household contact was reported for provinces which were more advanced in the de-escalation of physical distancing (e.g., PEI) at the time of survey completion; however, in provinces where physical distancing was still in place during the survey period (e.g., ON), approximately 20% of respondents were reporting non-household contacts in May 2020. Of the respondents who reported non-household contacts, 62% reported that this occurred once or twice in a 7-day period while almost 23% reported having non-household contacts more than 3 days out of the 7-day period prior to survey completion (Fig. 3b). Individuals in the youngest age group and those who reported an annual household income greater than $110,000 were more likely to have participated in an activity with someone outside their household (Table 5) compared with older respondents and those earning less than $60,000, respectively. Perceived risk of serious illness was associated with less interaction with individuals outside the household.

**Table 5:** Results of a multivariable logistic regression analysis assessing factors associated with engaging in an activity with non-household contacts in the 7 days prior to survey completion. Values are reported as adjusted odds ratios (95% confidence interval) and those in bold font are statistically significant (*p* < 0.05) (*n* = 4981)

Variable	Adjusted OR (95% CI)	*p* Wald’s test	*p* (L-R test)
Age category			< 0.001
**18–29 years**	**1.74 (1.39–2.18)**	**< 0.001**	
30–39 years	1.18 (0.95–1.48)	0.14	
40–49 years (referent)	-	-	
50–59 years	0.98 (0.78–1.24)	0.88	
60–69 years	1.22 (0.97–1.52)	0.09	
Over 70 years	0.90 (0.69–1.18)	0.46	
Household income of respondent			< 0.001
$0–$60,000 (referent)	-		
$60,001–$110,000	1.09 (0.93–1.28)	0.29	
**> $110,000**	**1.49 (1.25–1.78)**	**< 0.001**	
Unsure/prefer not to answer	1.03 (0.82–1.30)	0.80	
Perceived risk of COVID as a serious illness to self			0.006
No (referent)			
**Yes**	**0.83 (0.72**–**0.95)**	**0.006**	

Details of the regression model identifying factors associated with confidence in the ability to self-isolate with mild symptoms of COVID-19 are located in Table S2 of the Supplementary Materials.

## Discussion

At the time of data collection, Canadian provinces were in various stages of reopening the economy. Overall, the majority of participants reported risk perceptions and attitudes about the effectiveness of public health measures that are well aligned with scientific evidence and the reported ability to comply with such measures was high. However, there are differences in these indicators based on socio-demographic variables and context. If our collective priority is to maintain an open economy, we need to ensure that individuals are able to comply with public health measures that prevent and control transmission of the virus. The results from this study have identified a number of areas in which policies could help address issues of public adherence.

While confidence in the ability to comply with various public health measures was high, younger age groups, those in the paid workforce, and, in some cases, those with lower income were less likely to report confidence in the ability to comply. Individuals need to feel supported in complying with public health measures. Our findings of reduced confidence in ability to comply with public health measures are consistent with other research demonstrating that those with a low income (Wolf et al. 2020) and those in younger age groups (Cvetković et al. 2020) are less prepared in the event of illness. Compliance in the event of self-isolation or quarantine is at least partially dependent on preparedness and having the means to self-isolate; there is a need to develop supports for those who need to self-isolate but may not have the means to do so. Presenteeism risk (attending work while sick) was gendered, with more men than women anticipating that co-workers would expect them to continue to work while sick; however, responses did not differentiate between employer and co-workers. More individuals with less education or income were at risk of not being paid if they took sick leave. Presenteeism has been shown to be prevalent among occupations with high contact rates, including the care, welfare, and education sectors (Aronsson et al. 2000). Determinants of presenteeism include job insecurity, workplace performance indicators that include attendance rates, and limited entitlement to paid sick leave (Kinman 2019). Investigators in Israel demonstrated that paid sick time increases compliance with stay-home-when-sick policies from 57% with no compensation to almost 100% when compensation was assumed (Bodas and Peleg 2020). These findings highlight the need for a shift in workplace culture toward discouraging presenteeism and ensuring paid sick time.

Our finding that a majority of respondents with dependent children were responsible for childcare at the same time as maintaining employment when schools and daycares were closed due to the pandemic highlights the need for provincially co-ordinated plans with evidence-based infection prevention and control (IPAC) procedures for safe school functioning. There is mounting evidence that children are more susceptible and can transmit infection more readily than originally thought, potentially contributing substantially to community transmission (Hyde 2020). Recognition of transmission among children could lead to prolonged school closures in provinces heavily impacted by the pandemic. Statistics Canada estimates that there are more than 10 million families with children living in Canada (Statistics Canada 2020) and almost 70% of families with dependent children have two employed parents (Statistics Canada 2016b). Investing in robust IPAC procedures for schools will allow schools to remain open and parents to maintain employment.

An indication that public health messaging has been successful in many cases, we found that both the perceived ability to comply with public health measures and perceived effectiveness of such measures were very high. However, both indicators varied by age and gender, emphasizing the need for additional targeted messaging. The finding that perceived risk of serious illness increased with age group is consistent with past research (Bruine de Bruin 2020; He et al. 2020) and is in line with empiric estimates of illness risk in older individuals (Public Health Agency of Canada 2020). Perceived lack of risk in younger individuals was associated with poor compliance with public health measures and is consistent with a growing body of evidence demonstrating that male gender and younger age groups engage in more COVID-19 risk behaviours (Alsan et al. 2020; Seale et al. 2020). Younger adults tend to have larger contact networks than older adults (Mossong et al. 2008) which likely partially explains these results. Recent increases in cases of COVID-19 in adolescents and young adults have been attributed to greater mixing among this age group combined with lower adherence to physical distancing measures (Goldstein and Lipsitch 2020).

The evidence for the efficacy of non-medical masks for COVID-19 prevention continues to grow (Fisman et al. 2020; Konda et al. 2020; Lyu and Wehby 2020; Rodriguez-Palacios et al. 2020). While the survey question for mask use was not restricted to people who had left their household in the previous 24 hours, fewer than one third of respondents reported wearing a face mask in the 24 hours prior to survey completion. Mask use was associated with household composition and the strongest association was belonging to a household with more than two generations living together, likely reflecting concern for the safety of older individuals in households. Increased mask use in the youngest age group may reflect younger individuals working in essential service jobs at the time of the survey (e.g., grocery stores). As with other preventive measures, compliance with masks was more likely in individuals with greater self-perceived risk.

## Limitations

While every effort was made to ensure representativeness of the study population, we note several potential biases, including non-representativeness of the sample (a risk with any survey), the online nature of the survey, which limits participation to those who use the Internet, and self-report which introduces the potential for recall, response, and social desirability biases. The large sample size means that statistical significance is seen with small absolute differences. The results are consistent with the large body of research on risk attitudes and behaviours and, on their own, would add little new knowledge to the literature. However, governments and public health officials have asked Canadians to comply with extraordinary measures. The value of this study lies in its assessment of the ability to comply with these extraordinary measures during a pandemic given a variety of socio-demographic characteristics. Finally, knowledge about COVID-19 and recommended behaviours is changing rapidly. These data were collected in May 2020 during a time in which provinces were in different phases of public health de-escalation and indoor masking orders were not widespread, so these data are best interpreted as a snapshot in time.

## Conclusion

The results of this study highlight the need for the development of enhanced messaging and financial supports in order to further support improved compliance with public health measures. Work is needed to identify strategies and develop tools for targeted messaging to groups who are more likely to engage in risk behaviours, and social support is needed for lower income individuals to enable periods of self-isolation and childcare should they become ill, to permit schools to be open for in-person learning, and to discourage presenteeism. Taken together, such measures are likely to mitigate the impact of the COVID-19 pandemic in Canada.

## Supplementary Information

ESM 1 (DOCX 53 kb)

## Data Availability

Aggregated data are available upon request from the corresponding author.

## Code availability

Code is available upon request from the corresponding author.

## Declarations

The funders had no role in study design, data collection and analysis, decision to publish or preparation of the manuscript.
