# Supplementary material for: Socio-demographic disparities in knowledge, practices, and ability to comply with COVID-19 public health measures in Canada
Source: Can J Public Health. 2021 Mar 24;112(3):363–75. doi: 10.17269/s41997-021-00501-y (PMC7989685; doi:10.17269/s41997-021-00501-y)
Supplement: Supplementary file 1 — (DOCX 53 kb) [file 41997_2021_501_MOESM1_ESM.docx]

**Table S1.** Sociodemographic characteristics of survey respondents. Gender, Age Category, and Province of Residence are compared with the Canadian population 18 years and older, excluding the Territories.

|  | **Number of respondents (%)** | **2016 Census** |
| --- | --- | --- |
|  | **N = 4981** | **N = 28,040,360** |
| **Gender** |  |  |
| Male | 2444 (49.1%) | 13,619,150 (48.6%) |
| Female | 2520 (50.6%) | 14,421,140 (51.4%) |
| Other (e.g., trans, non-binary, two-spirit, gender-queer) | 17 (0.34%) | - |
| **Age Category** |  |  |
| 18-29 years | 781 (15.7%) | 5,344,675 (19.1%) |
| 30-39 years | 935 (18.8%) | 4,600,485 (16.4%) |
| 40-49 years | 802 (16.1%) | 4,600,310 (16.4%) |
| 50-59 years | 848 (17.0%) | 5,283,150 (18.8%) |
| 60-69 years | 977 (19.6%) | 4,253,465 (15.2%) |
| 70 + years | 638 (12.8%) | 3,958,275 (14.1%) |
| **Province of Residence** |  |  |
| Newfoundland | 76 (1.5%) | 519,716 (1.5%) |
| Prince Edward Island | 23 (0.5%) | 142,907 (0.4%) |
| New Brunswick | 82 (1.7%) | 747,101 (2.1%) |
| Nova Scotia | 160 (3.2 %) | 923,598 (2.6%) |
| Quebec | 1170 (23.5%) | 8,164,361 (23.3%) |
| Ontario | 1909 (38.3 %) | 13,448,494 (38.4%) |
| Manitoba | 189 (3.8%) | 1,278,365 (3.6%) |
| Saskatchewan | 160 (3.2%) | 1,098,352 (3.1%) |
| Alberta | 521 (10.5%) | 4,067,175 (11.6%) |
| British Columbia | 691 (13.9%) | 4,648,055 (13.3%) |
|  |  |  |
| **Risk Group and/or Pregnant (n = 4981)** |  | |
| Yes | 1620 (32.5%) | |
| **Education Level (n = 4981)** |  | |
| Secondary or less | 1192 (23.9%) | |
| College/Trades/Other qualification | 1896 (38.1%) | |
| University degree | 1893 (38.0%) | |
| **Employment Status (n = 4981)** |  | |
| Full-time | 1785 (35.8%) | |
| Part-time | 662 (13.3%) | |
| Self-employed | 251 (5.0%) | |
| Unemployed | 414 (8.3%) | |
| Working within the home | 129 (2.6%) | |
| Retired | 1428 (28.7%) | |
| Student | 215 (4.3%) | |
| Other | 97 (2.0%) | |
| **Respondent Unemployed Due to COVID (n = 414)** |  | |
| Yes | 167 (40.3%) | |
| **Household income (n = 4981)** |  | |
| $0-$60,000 | 1870 (37.5%) | |
| $60,001-$110,000 | 1575 (31.6%) | |
| > $110,000 | 1024 (20.6%) | |
| Unsure/Prefer not to answer | 512 (10.3%) | |
| **Size of Geographic Area of Residence (n = 4981)** |  | |
| Large city | 2106 (42.3 %) | |
| Medium sized city | 1399 (28.1%) | |
| Large town | 485 (9.7%) | |
| Small town | 611 (12.3%) | |
| Rural place | 380 (7.6%) | |

*Confidence in the ability to self-isolate with mild symptoms of COVID-19*

A multivariable logistic regression model was developed to identify factors associated with reporting confidence in the ability to self-isolate with mild symptoms of COVID-19. Univariable models were first assessed using a liberal p-value of less than 0.3 to determine eligibility for inclusion in the multivariable models. Variables included in the initial full model included age, gender, risk group status, size of geographic region of residence, household income, education level, employment status, household composition, household size, as well as two indicators of perceived risk of COVID-19 to self and one indicator of perceived risk of transmission to others. Household characteristics such as whether the household included children or a single parent, and whether the household had access to a 14-day stockpile of supplies were also included. In addition, workplace indicators such as access to paid sick leave, expectation to work while sick, and the ability to work remotely were included in the initial full model.

Individuals who reported an annual household income between $60,001 and $110,000 reported more confidence in the ability to self-isolate compared with those earning less **(Table S2).** Those who perceived COVID-19 to be a serious illness and those who considered themselves at risk of transmitting to others were more likely while those who considered themselves to be at risk of contracting the virus were less likely to report confidence to comply with this measure **(Table S2).** Job-related variables such as being a member of the paid workforce were less likely to report confidence to comply, however those who were able to work remotely and those who were not expected to work while sick were more likely to be confident. The only interaction detected showed that those older than 50 years of age reported greater confidence in the ability to self-isolate even when they did not have access paid sick leave compared the youngest age group with access to paid sick leave **(Table S2).** Taken alone, lack of access to paid sick leave was associated with a lower level of confidence to self-isolate. Lastly, individuals who reported access to enough food and supplies to last 14 days were 2.5 times as likely to be confident. Multicollinearity was not detected within any of the three regression models.

**Table S2.** Results of a multivariable logistic regression analysis assessing factors associated with reported confidence in the ability to self-isolate with mild symptoms of COVID-19. Values are reported as adjusted odds ratios (95% Confidence Interval) and those in bold font are statistically significant (p < 0.05) (n = 4981).

| **Variable** | **Adjusted OR (95% CI)** | **P Wald’s test** | **P (L-R test)** |
| --- | --- | --- | --- |
| Age Category |  |  | 1 |
| 18-29 years (referent) | - |  |  |
| 30-39 years | 0.94 (0.66 – 1.35) | 0.75 |  |
| 40-49 years | 1.40 (0.95 – 2.08) | 0.09 |  |
| **50-59 years** | **1.79 (1.18 – 2.71)** | **0.01** |  |
| **60-69 years** | **1.90 (1.23 – 2.92)** | **0.004** |  |
| **Over 70 years** | **4.34 (2.18 – 8.63)** | **<0.001** |  |
|  |  |  |  |
| Household income of respondent |  |  | 0.03 |
| $0 - $60,000 (referent) | - |  |  |
| **$60,001 - $110,000** | **1.41 (1.09 – 1.84)** | **0.009** |  |
| > $110,000 | 1.19 (0.89 – 1.58) | 0.24 |  |
| Unsure/prefer not to answer | 0.88 (0.62 – 1.25) | 0.47 |  |
|  |  |  |  |
| Perceived risk of COVID as a serious illness to self |  |  | <0.001 |
| No (referent) | **-** |  |  |
| **Yes** | **1.57 (1.26 – 1.96)** | **<0.001** |  |
|  |  |  |  |
| Perceived risk of COVID of contracting the virus |  |  | 0.01 |
| No (referent) | **-** |  |  |
| **Yes** | **0.72 (0.56 – 0.92)** | **0.01** |  |
|  |  |  |  |
| Perceived risk of COVID of transmitting to others |  |  | 0.02 |
| No (referent) | **-** |  |  |
| **Yes** | **1.34 (1.06 – 1.68)** | **0.01** |  |
|  |  |  |  |
| Employment status of respondent |  |  | <0.001 |
| Unemployed, Student, Retired, Work w/in Home (referent) | **-** |  |  |
| **Employed FT, PT, Self Employed** | **0.48 (0.37 – 0.62)** | **<0.001** |  |
|  |  |  |  |
| Perceived effectiveness of reducing contacts |  |  | <0.001 |
| **Perceived that reducing contacts would be effective** | **5.57 (4.18 – 7.41)** | **<0.001** |  |
|  |  |  |  |
| Ability to work remotely |  |  | <0.001 |
| **Respondent has a job that is possible to do remotely** | **1.54 (1.25 – 1.90)** | **<0.001** |  |
|  |  |  |  |
| Expectation to work when sick |  |  | <0.001 |
| **Respondent would not be expected to work when sick** | **1.96 (1.57 – 2.44)** | **<0.001** |  |
|  |  |  |  |
| Paid sick leave |  |  | 1 |
| **Respondent does not have access to paid sick leave** | **0.42 (0.25– 0.69)** | **<0.001** |  |
|  |  |  |  |
| 14- day stockpile |  |  | <0.001 |
| **Respondent has access to food and supplies to last 14 days** | **2.59 (2.10 – 3.19)** | **<0.001** |  |
|  |  |  |  |
| Interaction between age category and access to paid sick leave |  |  | 0.002 |
| 18-29 years with paid sick leave (referent) | **-** |  |  |
| 30-39 years with no paid sick leave | 0.99 (0.521– 1.92) | 0.99 |  |
| **40-49 years with no paid sick leave** | **2.60 (1.33 – 5.08)** | **0.01** |  |
| **50-59 years with no paid sick leave** | **2.14 (1.01 – 4.50)** | **0.05** |  |
| **60-69 years with no paid sick leave** | **2.97 (1.37 – 6.45)** | **0.01** |  |
| **Over 70 years with no paid sick leave** | **3.16 (0.78 – 12.82)** | **0.11** |  |

**Survey Instrument**

1. What is your age?
2. Are you...

- A man
- A woman
- In another way (eg. Trans, non-binary, two-spirit, gender-queer)

1. Which province do you currently live in?

- Newfoundland and Labrador
- Prince Edward Island
- New Brunswick
- Nova Scotia
- Quebec
- Ontario
- Manitoba
- Saskatchewan
- Alberta
- British Columbia

1. What is your postal code? (Please use upper case letters only e.g. A0A 0A0)
2. What is the highest level of school you have completed, or the highest degree you have received?

- Less than secondary/high school
- Secondary/high school
- Non-trade qualification
- Trade qualification
- Associate Diploma
- Undergraduate Diploma
- Bachelor Degree (including Honours)
- Postgraduate Degree or Postgraduate Diploma
- Other (specify) ________________________________________________

1. Which of the following best describes your current situation. Are you:

- Employed part-time
- Employed full-time
- Self-employed
- Unemployed and looking for work
- Unemployed and not actively looking for work
- Working within the home
- Retired
- Student
- Other (specify) ________________________________________________

1. Are you unemployed as a result of the COVID-19 pandemic?

- Yes
- No

1. What is your occupation? If retired or unemployed, please indicate the category closest to your previous occupation.

- Management
- Professional
- Technical or trade
- Community or Personal Service
- Clerical or Administrative
- Sales
- Machinery Operation or Driving
- Labour
- Other (specify) ________________________________________________

1. Do you usually attend daycare, school, college, or university?

- Yes
- No

1. Are you pregnant?

- Yes
- No

1. Are you in a high-risk group for which the annual seasonal influenza vaccine would usually be recommended by the Public Health Agency of Canada? These conditions include individuals with chronic respiratory disease, chronic heart disease, chronic kidney disease, chronic liver disease, chronic neurological disease, diabetes (all types), cancer, immunosuppression, dysfunction of the spleen, and/or BMI > 40.

- Yes
- No

1. Please write the nickname of each person in your household (excluding yourself). These people should live at the same address and share a kitchen with you. If you live by yourself, please move to the next question. Note that these nicknames are only needed to make it easier for you to complete the survey, so please pick a nickname that will help you identify each contact. Nicknames are not visible to anyone outside of this survey.

- Household member 1 ________________________________________________
- Household member 2 ________________________________________________
- Household member 3 ________________________________________________
- Household member 4 ________________________________________________
- Household member 5 ________________________________________________
- Household member 6 ________________________________________________
- Household member 7 ________________________________________________
- Household member 8 ________________________________________________

1. What is this person's age?
2. To your knowledge, which of the following describes how this person thinks of themself?

- Male
- Female
- In another way (eg. Trans, non-binary, two-spirit, gender-queer)
- Prefer not to say

1. What is the highest level of school this person completed, or the highest degree received?

- Less than secondary/high school
- Secondary/high school
- Non-trade qualification
- Trade qualification
- Associate Diploma
- Undergraduate Diploma
- Bachelor Degree (including Honours)
- Postgraduate Degree or Postgraduate Diploma
- Other (specify) ________________________________________________

1. Which of the following best describes this persons's current situation. Are they:

- Employed part-time
- Employed full-time
- Self-employed
- Unemployed and looking for work
- Unemployed and not actively looking for work
- Working within the home
- Retired
- Student
- Other (specify) ________________________________________________

1. Is this person unemployed as a result of the COVID-19 pandemic?

- Yes
- No

1. What is this person's occupation? If retired or unemployed, please indicate the category closest to their previous occupation.

- Management
- Professional
- Technical or trade
- Community or Personal Service
- Clerical or Administrative
- Sales
- Machinery Operation or Driving
- Labour
- Other (specify) ________________________________________________

1. Does this person usually attend daycare, school, college, or university?

- Yes
- No

1. To your knowledge, is this person pregnant?

- Yes
- No

1. Is this person in a high-risk group for which the annual seasonal influenza vaccine would usually be recommended by the Public Health Agency of Canada? These conditions include individuals with chronic respiratory disease, chronic heart disease, chronic kidney disease, chronic liver disease, chronic neurological disease, diabetes (all types), cancer, immunosuppression, dysfunction of the spleen, and/or BMI > 40.

- Yes
- No

1. To what extent do you agree or disagree with each of the following statements?

|  | Strongly agree | Somewhat agree | Neither agree nor disagree | Somewhat disagree | Strongly disagree | Don't know |
| --- | --- | --- | --- | --- | --- | --- |
| Coronavirus would be a serious illness for me |  |  |  |  |  |  |
| I am likely to catch coronavirus |  |  |  |  |  |  |
| If I don’t follow the government’s advice, I might spread coronavirus to someone who is vulnerable |  |  |  |  |  |  |

1. How effective, if at all, do you think each of the following are at slowing the spread of coronavirus?

|  | Very effective | Fairly effective | Not very effective | Not at all effective | Don't know |
| --- | --- | --- | --- | --- | --- |
| Reducing the number of people you meet |  |  |  |  |  |
| Staying at home for 14 days if you have a mild symptom such as a mild cough |  |  |  |  |  |
| Staying at home for 14 days if you have more severe symptoms such as a severe cough or a high temperature |  |  |  |  |  |
| Avoiding crowded places |  |  |  |  |  |
| Staying at home for 14 days if anyone other than yourself in your household has mild symptoms such as a mild cough |  |  |  |  |  |
| Staying at home for 14 days if anyone other than yourself in your household has severe symptoms such as a cough or a high temperature |  |  |  |  |  |
| School closures |  |  |  |  |  |
| Closing bars, restaurants, cinemas etc. |  |  |  |  |  |

1. How confident are you, if at all, that if you wanted to you could...?

|  | Very confident | Fairly confident | Not very confident | Not at all confident | Don't know |
| --- | --- | --- | --- | --- | --- |
| Reduce the number of people you meet |  |  |  |  |  |
| Stay at home for 14 days if you have a mild symptom such as a mild cough |  |  |  |  |  |
| Stay at home for 14 days if you have more severe symptoms such as a severe cough or a high temperature |  |  |  |  |  |
| Avoid crowded places |  |  |  |  |  |
| Stay at home for 14 days if anyone other than yourself in your household has mild symptoms such as a mild cough |  |  |  |  |  |
| Stay at home for 14 days if anyone other than yourself in your household has severe symptoms such as cough or a high temperature |  |  |  |  |  |
| Not use public transport |  |  |  |  |  |

1. To what extent do you agree or disagree with each of the following statements?

|  | Strongly agree | Somewhat agree | Neither agree nor disagree | Somewhat | Strongly disagree | Don't know |
| --- | --- | --- | --- | --- | --- | --- |
| Other people I work with expect me to work, even when I am ill |  |  |  |  |  |  |
| If I could not work because of coronavirus, I would still get paid |  |  |  |  |  |  |
| If I had to isolate myself for 14 days because of coronavirus, someone else would be able to look after my children |  |  |  |  |  |  |
| If I had to isolate myself for 14 days, this would cause problems for other people who I don’t know |  |  |  |  |  |  |
| I have enough food and supplies to last for 14 days, if I had to isolate myself |  |  |  |  |  |  |

1. Is it possible for you to do your job from home?

- Yes
- No

1. Approximately how often did you **work from your home** rather than working from your usual office or worksite in the past year?

- Daily
- 3-5 times a week
- 1-2 times a week
- 1-3 times a month
- 6-11 times a year
- 3-5 times a year
- 1-2 times a year
- Never

1. In the **last 7 days** have you (or any member of your household):

|  | Yes | No | Not Applicable | Prefer not to answer |
| --- | --- | --- | --- | --- |
| Participated in any activity (indoors or outdoors) with 1 or more people from outside of your household (this includes visits/meals with friends and/or other family members from outside your household, playdates etc.) |  |  |  |  |
| Worked from home/engaged in telework instead of physically going to your workplace |  |  |  |  |

1. How many days out of the **past 7 days** have you (or any member of your household):

|  | 0 | 1 | 2 | 3 | 4 | 5 | 6 | 7 |
| --- | --- | --- | --- | --- | --- | --- | --- | --- |
| Participated in any activity (indoors or outdoors) with 1 or more people from outside of your household (this includes visits/meals with friends and/or other family members from outside your household, playdates etc.) |  |  |  |  |  |  |  |  |
| Worked from home/engaged in telework instead of physically going to your workplace |  |  |  |  |  |  |  |  |

1. When daycares and schools were closed due to coronavirus (COVID-19) who looked after the children in your household? (Choose all that apply)

- A parent, who is unemployed
- A parent, who works within the home
- A parent, who was working from home
- A parent, who works part-time
- A parent, who took vacation leave
- A parent, who took unpaid leave
- A sibling
- Grandparent(s)
- A baby sitter, childminder, au pair or nanny (paid)
- A baby sitter, childminder, au pair or nanny (unpaid)
- A neighbour, friend, uncle, or aunt
- Other (please specify) ________________________________________________

1. Are the following people you selected members of your household (i.e. lives at the same address and shares a kitchen)?

|  | Yes | No | Prefer not to say |
| --- | --- | --- | --- |
| A parent, who is unemployed |  |  |  |
| A parent, who was working from home |  |  |  |
| A parent, who works part-time |  |  |  |
| A parent, who took vacation leave |  |  |  |
| A parent, who took unpaid leave |  |  |  |
| A sibling |  |  |  |
| Grandparent(s) |  |  |  |
| A baby sitter, childminder, au pair or nanny (paid) |  |  |  |
| A baby sitter, childminder, au pair or nanny (unpaid) |  |  |  |
| A neighbour, friend, uncle, or aunt |  |  |  |
| Other (please specify) |  |  |  |

1. Did you use a face mask of any type yesterday?

- Yes
- No

1. For how long did you wear a face mask in total? (Provide an approximation of the total duration **in minutes**).
2. Where did you use your face mask? (Select all that apply)

- Everywhere outside my house
- When walking on the street
- When cycling
- On public transportation
- In supermarkets/stores
- At home
- At work/school/college/university
- Other (please specify) ________________________________________________

1. Did you travel on any public transportation yesterday? (select all)

- No
- Train/subway
- Bus/streetcar
- Taxi, Uber, or similar ride-sharing service
- Airplane

1. Approximately how long did you spend on public transportation in total yesterday? (in minutes)
